# Decreasing Stigmatization: Reducing the Discrepancy Between “Us” and “Them”. An Intervention for Mental Health Care Professionals

**DOI:** 10.3389/fpsyt.2019.00243

**Published:** 2019-05-31

**Authors:** Kim Helmus, Iris Kleine Schaars, Hansje Wierenga, Elise de Glint, Jim van Os

**Affiliations:** ^1^Arkin, Amsterdam, Netherlands; ^2^Department of Psychiatry, Amsterdam UMC (AMC), Amsterdam, Netherlands; ^3^GGNet, Warnsveld, Netherlands; ^4^Mentaal Beter, Almere, Netherlands; ^5^FortaGroep, Rotterdam, Netherlands; ^6^Utrecht University Medical Centre, Utrecht, Netherlands

**Keywords:** stigma and discrimination, psychiatry, judgement (error), ingroup outgroup, diminishing stigma, mental health care professionals, workshop, intervention

## Abstract

**Objective:** Stigmatization has negative consequences for people with mental health disorder diagnosis. Studies indicate that professionals have stigmatizing attitudes and behavior towards clients. Continuum beliefs are associated with less stigmatizing attitudes. The effect of a workshop to diminish stigmatizing attitudes and to enhance continuum beliefs is examined.

**Method:** A total of 202 mental health professionals from (Functional) Assertive Community Treatment [(F)ACT] teams were randomly assigned to a workshop or a waiting list control group. Stigmatizing attitudes and continuum beliefs were assessed in both conditions at baseline and follow-up.

**Results:** Compared to baseline, the workshop group showed an increase on continuum beliefs. However, there was no effect of the intervention on stigmatizing attitudes. Contrary to the expectations, stigmatizing attitudes increased in the waiting list condition.

**Conclusion:** Communicating the continuity aspect can be valuable in decreasing the “us and them” discrepancy between professionals and people with mental health disorders. Further research on continuum beliefs is needed.

## Introduction

In all countries, societies are confronted with issues that are perceived as aversive, such as crime, failure, weakness, and physical or psychological problems (1). Often, these issues evoke emotional responses such as anger, fear, or pity (2). As described by Dijker and Koomen (3), when these issues occur, or when people expect them to occur, they tend to respond with one of the following strategies: repair, tolerance, or stigmatization (4). Repair aims to change the observed or imagined deviance of the person by using care, treatment, or punishment. Tolerance involves trying to ignore one’s responses to the deviant person. Stigmatization involves the social rejection and exclusion of the person. The present research focuses on the last strategy (stigmatization) within the context of mental health care.

Stigmatization involves a process in which a condition is observed by an individual or group and is seen as deviant, evoking negative emotions and thoughts (5, 6). This observation may entail a current, former, or observer-imagined condition (3). The cognitions and feelings triggered by this observation can lead to labelling, discrimination, prejudice, separation, stereotyping, and status loss of the stigmatized individual (7). Stigmatization can be expressed in different ways, depending on (8), for example, the situation, context, previous experiences, values, and goals of people (9).

One factor influencing the process of stigmatization is the tendency of people to divide the world into categories (10). By doing so, people simplify the complexity of the world around them, which allows them to use their cognitive resources for other tasks (11). When people are categorized into groups, differences within groups tend to be minimized (ingroup) and differences between groups are exaggerated (outgroup) (12, 13). People prefer ingroup members, “us,” over outgroup members, “them.” This process is called intergroup bias (14, 15), and it can lead to exclusion, discrimination, and inequality within society and/or relationships (3, 6, 16–21).

People with mental health problems often suffer from the consequences of intergroup bias. They are stigmatized, sometimes despite recovery from their problems (18, 22–24). Common underlying cognitions are beliefs of potential danger, incompetence, “being less useful to society,” and being responsible for their problems (22, 24–26). The effects of stigmatization can be harmful for stigmatized individuals, as these effects can increase self-stigmatization and mental health problems, and decrease self-esteem, feelings of hope, quality of life, and willingness to seek help (27, 28).

People with mental health problems experience stigmatization in mental health care facilities approximately to the same extent as within the general population (29–31). Almost a quarter (22%) of the total experience of stigmatization is being attributed to contact with mental health care professionals (29, 32). Reavley et al. (33) found in their study (*N* = 6019) measurements on stigmatizing attitudes among mental health care professionals to be as strong as those within the general population. The experience of being stigmatized by mental health care professionals negatively influences recovery (27, 34–36).

As mentioned above, one of the causes of stigmatization is the categorization of “us” (mental health care professionals) versus “them” (clients with mental health problems). Yet, the distinction between people with and without mental health problems seems to be arbitrary. All symptoms of mental health disorders occur within the whole population at least to some extent and vary in intensity over time (37–40). Therefore, psychological problems can also be seen on a continuum (21, 40, 41). When mental health care professionals see people with mental health problems as members of their own group, when they are convinced that symptoms of mental health disorders are a severe variant of what is normal, and when they see that the intensity of problems can vary on a continuum over time, they tend to stigmatize less (21, 42). This is called “continuum belief”; people with mental health disorders are then no longer considered to be an outgroup. As a consequence, the likelihood of fearful reactions and social distance towards people with mental health disorders decreases and prosocial behavior is promoted (42–45).

In order to decrease the gap between “us” and “them,” and thereby reducing stigmatization, two methods of interventions have proven to be effective. The first one is direct, personal contact based on equality between people from the “ingroup” and people from the “outgroup”, in this study, mental health care professionals, and people with mental health problems. The second method is education wherein prejudices are invalidated (46). Research has shown the strategy of a contact intervention based on equality to have the most impact (47, 48).

To our knowledge, there has not yet been much research on well-targeted interventions to reduce stigmatization within mental health care. Hence, the present study examines the effect of a recently developed contact-based workshop that focuses on breaking the barriers between “us” and “them.” The intervention is structured, small-scaled, and based on equality. Interaction between ingroups and outgroups is most effective when contact is supported by an authority (e.g., a trainer/psychologist) (14). Positive interpersonal communication, shared goals, cooperation, and equal status between people facilitate this process (24). The present study tries to answer the question whether the intervention results in a decrease of stigmatizing attitudes and an increase of continuum beliefs among mental health care professionals.

## Methods

### Participants

**Figure 1:**
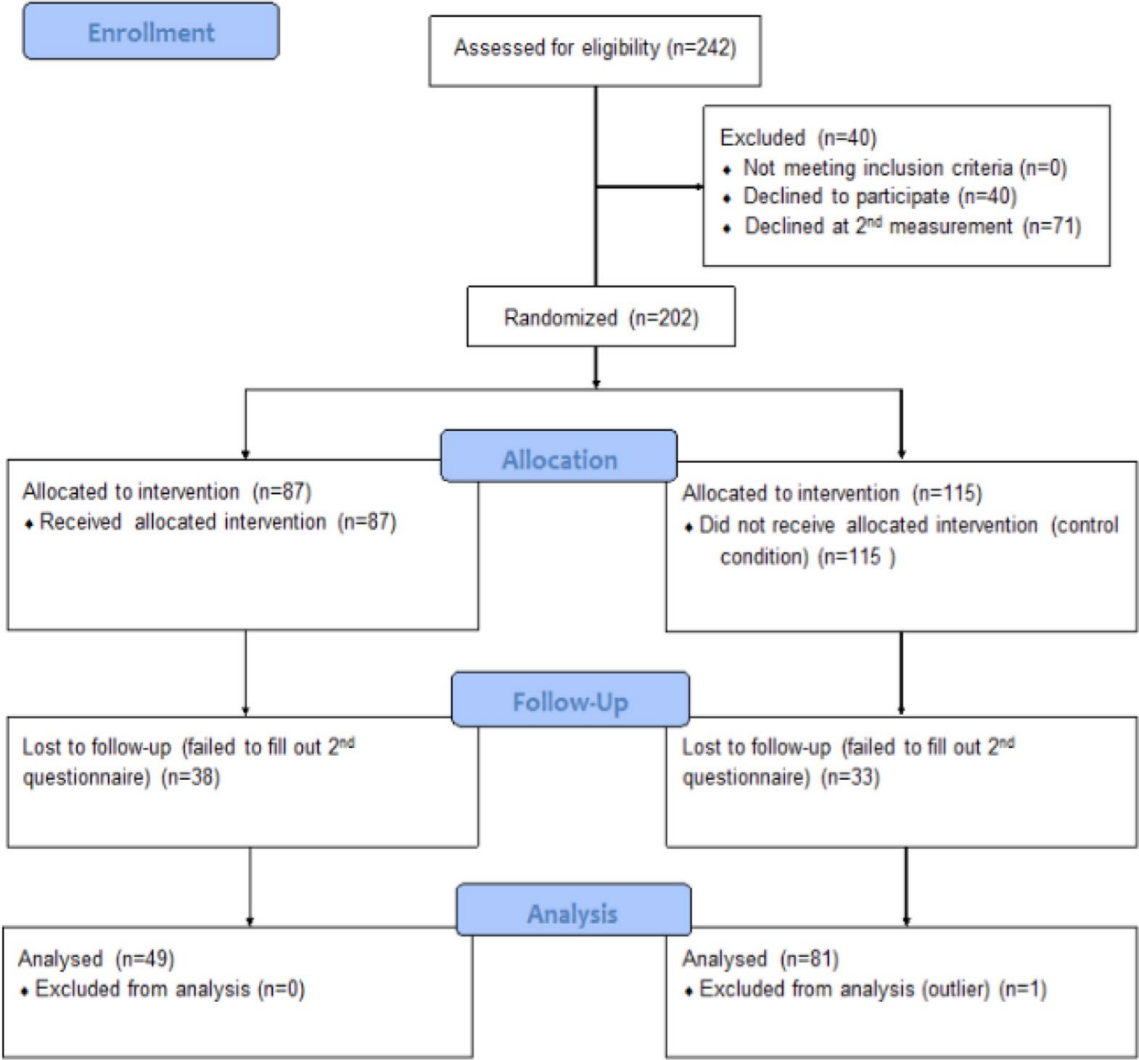
Interaction effect between time × condition on CBQ Total.

This study was aimed at mental health care professionals in the Netherlands. All professionals were recruited from (Functional) Assertive Community Treatment ([F]ACT) teams. (F)ACT teams use a standardized set of interventions and guidelines and serve patients with severe mental illness. Participants were approached through the managers of the teams of four mental health care institutions. Twenty-five out of 30 managers were willing to participate in the study; **Figure 1** shows the flow diagram of study enrollment. The teams were randomly assigned to either an experimental condition (EC) in which a workshop was offered or a control condition (CC). The workshop was offered to the professionals in the CC after the follow-up measures. In both conditions, participants were asked to fill out a questionnaire at baseline and at follow-up after 1 month. Participants were excluded when less than 90% of their questionnaire was completed. Every member of the team decided individually if they wanted to take part. In total, 202 mental health workers agreed to participate.

At baseline (T1), 87 participants in the EC and 115 in the CC completed the questionnaire. At 1-month follow-up (T2), 49 and 82 participants completed the questionnaire, respectively. There was a dropout of 71 participants, 37 (18.3%) in the EC and 34 (16.8%) in the CC. Demographic variables did not show large or significant differences between conditions or over time. The age of the professionals varied from 19 to 64 years. **Table 1** demonstrates the sex and average age of participants.

**Table 1:** Demographic characteristics of participants on T1 and T2.

	Age		Sex	
	T1	T2	T1	T2
Experimental condition	*M* = 43.8 *SD* = 12.1 N = 87	*M* = 42.9 *SD* = 11.7 N = 49	Male = 33 (37.9%) Female = 51 (58.6%) Missing = 3 (3.4%) N = 87	Male = 19 (38.8%) Female = 29 (59.2%) Missing = 1 (2.0%) N = 49
Control condition	*M* = 46.74 *SD* = 10.3 N = 115	*M* = 46.5 *SD* = 10.8 N = 82	Male = 37 (32.2%) Female = 76 (66.1%) Missing = 2(1.7%) N = 115	Male = 27 (32.9%) Female = 53 (64.6%) Missing = 2 (2.4%) N = 82
Total	*M* = 45.5 *SD* = 11.2 N = 202	*M* = 45.2 *SD* = 11.2 N = 131	Male = 70 (34.7%) Female = 127 (62.9%) Missing = 5 (2.5%) N = 202	Male = 46 (35.1%) Female = 82 (62.6%) Missing = 3 (2.3%) N = 131

M, mean; SD, standard deviation.

### Procedure

Prior to the research, ethical approval was obtained from the Ethics Committee of the Faculty of Social Sciences of Utrecht University (The Netherlands). A date was set with every team from the EC for the workshop to take place. The flowchart of inclusion is shown in **Figure 1**.

Two weeks prior to the workshop, the members of the teams in both the EC and CC received an informed consent, information about the workshop, the study, and the questionnaire. The questionnaire consisted of questions regarding demographic characteristics, stigmatizing attitudes (see the section Stigmatization), and continuum beliefs (see the section Continuum Beliefs). Furthermore, participants in the EC were asked to invite one or more of their clients to voluntarily join the team for the workshop. Clients received a briefing with information about the workshop. Approximately 1 month after the workshop took place, the professionals were asked to fill out the questionnaire once again. Subsequently, the participants received a debriefing. All data were processed anonymously.

### Materials

#### Workshop

The workshop, which was developed by the NGO *Wat Doe Jij?* (What Do You Do)?, was led by licensed and experienced psychologists and people with lived experience of mental disorders working in mental health care. The workshop consisted of the sharing of experiences with mental difficulties and stigmatization, education (e.g., a short movie) to increase awareness about the impact of stigmatization, joint effort/small group assignments (e.g., awareness and compassion exercises, storytelling, and cognitive interventions on stigmatizing beliefs), and a group discussion about enhancing acceptance and understanding of psychological difficulties. The duration of the workshop was two hour, and the group size varied from 4 to 18 professionals. The aim was to even the number of professionals and clients in a workshop (ratio score). No data were collected from clients. During the workshop, a minimum of one researcher was present to rate protocol compliance (protocol score), using a standardized manipulation check. Also, the client/mental health care professional ratio was evaluated (ratio score).

#### Stigmatization

Stigmatization (exclusion based on the process of emotional, cognitive, and behavioral aspects in social interaction) was measured with a focus on stigmatizing attitudes (i.e., the cognitive aspect). In order to assess stigmatizing attitudes, the Dutch version of the Mental Illness Clinicians’ Attitude scale (MICA) (49; Dutch translation: 50) was used. The MICA is a self-report questionnaire of 16 items, which can be rated on a six-point Likert scale (1 = fully agree, 6 = fully disagree). A higher score indicates a more stigmatizing attitude. According to Gras et al. (50), the Dutch version has an acceptable reliability (α = .73) and face validity. Reliability in the present study, however, was poor (α = .51). Three items were removed (3, 12, and 24) and 13 additional items were added. This improved the reliability to an acceptable level (α = .79). Moreover, the questionnaire contained linguistic errors and appeared to be very vulnerable to social desirability. Linguistic errors were corrected.

#### Continuum Beliefs

To assess continuum beliefs, the Continuum Beliefs Questionnaire (CBQ) (42) was used. This instrument consists of 16 items that were translated to Dutch according to the forward–backward method. All items were rated on a six-point Likert scale. A higher score indicates more continuum beliefs. Face validity and internal consistency of the original questionnaire are good (42). Three items were added to the translation from the questionnaires of Schomerus et al. (21), Peters et al. (41), and van Os et al. (40). In the current study, reliability was good (α = .80).

### Statistical Analyses

Data were analyzed using the Statistical Program for Social Sciences (SPSS), version 22. A repeated-measures ANOVA for independent groups was used, in order to identify the interaction effect of time (2) × condition (2) on stigmatizing attitudes and continuum beliefs (dependent variables).

## Results

### Hypotheses Concerning the Workshop

The first hypothesis was that stigmatizing attitudes among professionals who attended the workshop would decrease compared to the professionals in the control condition. This was analyzed by means of repeated-measures ANOVA. **Table 2** demonstrates the mean scores on the questionnaires. To test for main effects on MICA total scores on T1 and T2 in both conditions, two paired samples *t* tests were executed. The mean MICA total score in the EC before the workshop (*M* = 2.04, *SD* = .39) did not significantly differ from the total mean score after the workshop (*M* = 1.97, *SD* = .40; *t*[48] = 1.54, *p* = .13). However, in the CC, the mean MICA total score at T1 (*M* = 2.07, *SD* = .41) was significantly lower than the total mean score at T2 (*M* = 2.15, *SD* = .42; *t*[81] = −2.39, *p* = .02).

**Table 2:** MICA Total and CBQ Total per condition.

MICA						

	Control condition		Experimental condition		Total	
	T1	T2	T1	T2	T1	T2
N	82	82	49	49	131	131
M	2.07	2.15	2.04	1.97	2.06	2.08
SD	.41	.42	.39	.40	.40	.42
CBQ						
	Control condition		Experimental condition		Total	
	T1	T2	T1	T2	T1	T2
N	81	81	47	47	129	130
M	4.32	4.33	4.39	4.63	4.34	4.45
SD	.56	.51	.46	.46	.52	.53

On T1, one outlier was identified and excluded from the analysis. MICA Total = adjusted MICA + items developed by the researchers, CBQ Total = CBQ + added items.

**Figure 2:**
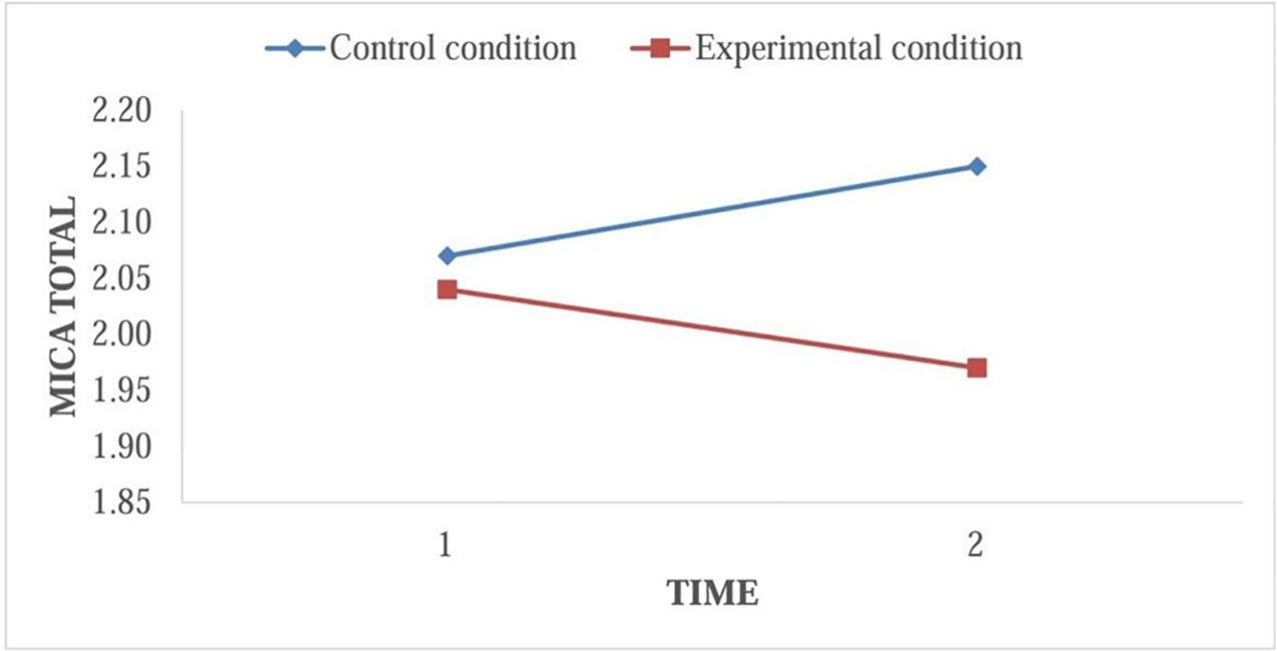
Interaction effect between time × condition on MICA Total.

A small interaction effect between time and condition on stigmatizing attitudes was found (*F*[1, 128] = 7.20, *p* < .01, partial *η* *^2^* = .05). However, as the paired samples *t* tests demonstrated, the interaction effect was not in the direction as predicted by the hypothesis, as the interaction was due to a small decline in the EC and a small increase in the CC (**Figure 2**).

### Correlations Between Protocol Score and/or Ratio Score and Stigmatizing Attitudes on T2

It was analyzed whether the protocol (compliance to protocol) and/or ratio score (rate health care professionals/cliënts) was correlated to stigmatizing attitudes on. This was confirmed for both scores (protocol score: *r* = −.25, *p* < .01; ratio score: *r* = −.28, *p* < .01). A higher score on one of these variables was associated with a lower MICA total score on T2. When controlling for these covariates, the interaction effect between time and condition disappeared (*F*[1, 123] = .27, *p* = .61).

### Hypotheses Concerning Continuum Beliefs

**Figure 3:**
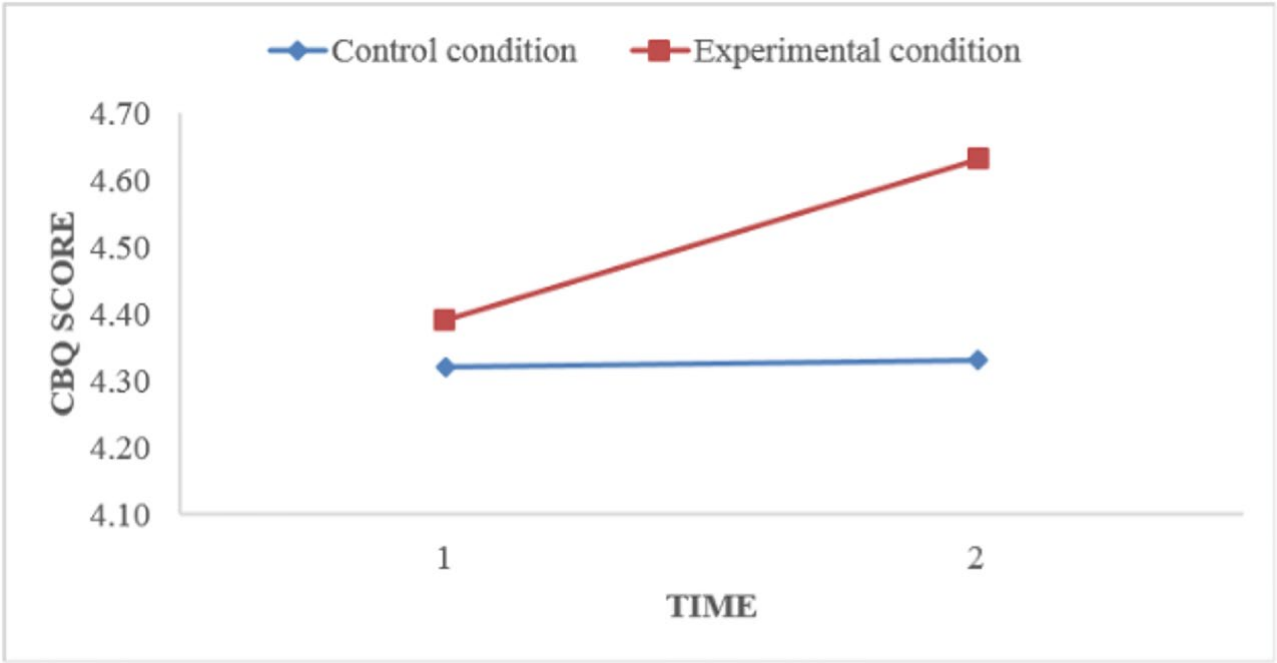
Effect of the workshop on continuum beliefs.

To compare the mean CBQ scores at T1 and T2 in both conditions, the same procedure was followed. The mean CBQ score in the EC before the workshop (*M* = 4.39, *SD* = .46) was significantly lower than the mean score after the workshop (*M* = 4.63, *SD* = .46; *t*[46] = −4.60, *p* < .01). In the CC, the mean CBQ score at T1 (*M* = 4.32, *SD* = .56) did not differ significantly from the mean score as shown in **Figure 3** at T2 (*M* = 4.33, *SD* = .51; *t*[80] = −.30, *p* = .77).

## Discussion

To the best of our knowledge, this has been the first study to examine the effects of an intervention focused on diminishing stigmatization by mental health care professionals towards people with mental health problems, where professionals together with clients receive a workshop. The intervention aimed at stimulating continuum beliefs (the belief that mental health problems are common and can be seen on a continuum of normal experiences) (42, 51). Stronger continuum beliefs are known to be associated with lesser stigmatizing attitudes (21).

Contrary to expectations, the workshop of this intervention study did not lead to a substantial reduction of stigmatizing attitudes. The workshop did, however, result in increased continuum beliefs. Another remarkable result is that stigmatizing attitudes increased within the control condition. These findings raise the question why the intervention effects continuum beliefs and not stigmatizing attitudes. Additionally, how can the increase of stigmatizing attitudes in the control condition be explained?

The results suggest that the workshop does not influence stigmatizing attitudes. Furthermore, it is questionable if at all a workshop is needed while very low scores on stigmatizing attitudes at baseline were found. Possibly, stigmatizing attitudes of the MHCP are not as common as presumed. However, the low scores on stigmatizing attitudes are not consistent with previous findings on stigmatizing attitudes among 1,522 health care workers (33). Stigmatizing attitudes of the health care professionals in the research of Reavley et al. (33) were found to be more present and even comparable to the stigmatizing attitudes of 5,019 members of the general population. Moreover, in a descriptive study by Gras et al. (50), the average score on the MICA in Dutch MHCP was higher than the average score found in the presented research.

Something that could explain the low scores on stigmatizing attitudes is that, in this study, participants were aware that stigmatization reduction was one of the aims of the intervention. This was not the case in the research by Reavleys et al. (33) and Gras et al. (50), which were descriptive studies. This “awareness of the aim of the intervention” could have increased the likelihood of social desirability behavior, a well-known phenomenon in stigma and discrimination research (52). In addition, research assistants reported that participants expressed aversion while filling out the questionnaire about stigmatizing attitudes (MICA). On some of the forms, remarks were made by the subjects: “wat een belachelijke vragen” (“what a ridiculous questions”). Items like “People with schizophrenia should not be allowed to work” are prone to trigger moral cognitions and the tendency to suppress negative responses (53). The subjects, due to their professional role, are expected to be helpful and non-judgemental towards the client group to whom they are offering their care. This can also have increased the likelihood of social desirability to occur (54). In the process of this study, strong doubts arose concerning the generalizability and validity of the MICA in (intervention) research due to the former described findings and due to the fact that the MICA only takes into account the cognitive aspect of stigmatization and not the behavioral and emotional aspect. Other, more sensitive and validated instruments are needed in order to learn and get a reliable image of the prevalence of stigmatization among MCHP.

A possible explanation for the remarkable finding of the increase of stigmatization within the control condition is described by Wegner (55) and is called the Ironic Process Theory. Where the subjects within the experimental condition were offered a workshop to diminish their stigmatizing attitudes, the subjects within the control condition had to wait after filling in the MICA questionnaire. In the day-to-day life of the MHCP, stigmatizing attitudes remain subconscious, partly because of negative associations and moral opinions about stigmatization. After having to answer the questions, the beliefs became overt. As there was no intervention offered to cope with these “unwanted beliefs”, it is possible that the subjects subconsciously tried to suppress their beliefs and thereby paradoxically made them even more likely to surface. Beliefs can grow stronger when people become more aware of them (56) or try to suppress them (55). This indicates that one should be careful using questionnaires about stigmatization without offering tools to cope with stigmatizing attitudes. Opposed to stigmatizing attitudes, continuum beliefs remained unchanged in the control condition. This might indicate that measuring stigmatizing attitudes is more prone to the Ironic Process than measuring continuum beliefs, which seems to be a more “neutral” concept and therefore does not trigger the tendency to suppress. It might be valuable in future research not so much to focus on stigmatizing attitudes or behavior but to focus on other variables that are closely related, such as continuum beliefs or destigmatizing behavior.

Within the experimental condition, the results show an increase in continuum beliefs. Apparently, the workshop was effective in increasing continuum beliefs. Perceiving that a person with mental problems is similar to ourselves may reduce social distancing by the public, which might be a more effective way of improving acceptance and reducing the “us and them” barrier (21, 57). Thus, the workshop may lend itself well to increase continuum beliefs. Stimulating continuum belief as a means to oppose perceived separation between “us” and “them” has been proven useful in destigmatizing interventions in studies by Wiesjahn et al. (58), in which a continuum belief intervention was consistently associated with lower stereotypes, less fear, and decreased desire for social distance. Schomerus et al. (21) showed similar results.

## Limitations and Future Research

In this study, the behavioral and emotional aspects of stigmatization were not taken into account, which makes it very difficult to truly understand the prevalence of stigmatization of the MHCP before and after the workshop. In future research, other, more sensitive instruments are needed to measure the full (cognitive, behavioral, and emotional) process of stigmatization. Furthermore, instruments that are less vulnerable to social desirability and that do not trigger stigmatizing attitudes when no intervention is being offered after measuring are needed. As mentioned above, it might be interesting to focus on the continuum aspect or the desired behavior instead of stigmatizing attitudes and behaviors. Also, one important shortcoming is that no qualitative or quantitative measurements were taken from the clients who were voluntarily participating in the workshop. This was due to limited available time, and the researchers chose to focus on MHCP. In hindsight, this was regrettable because research assistants mentioned that the clients who participated often said that they found the workshop a very valuable tool to reduce their self-stigmatization. It might be interesting in future research to have a closer look at the effects of the workshop on (self)stigmatization on an individual level. Furthermore, it could be valuable to look at acceptance and commitment (act) therapy as a theoretical framework to support this workshop and for future (self)stigmatization research (59, 60). The workshop in this study contains interventions that are in line with the theory behind act-therapy. Future research could also look at the role of continuum beliefs and stigmatizing attitudes in client–MHCP interaction/therapeutic relationship.

## Conclusion

The present research demonstrates that stigmatizing attitudes of the MHCP were low at baseline and did not decrease after receiving the workshop. In the control condition, stigmatizing attitudes increased. Other, more sensitive instruments are needed in order to measure stigmatizing attitudes. Continuum beliefs did increase after the workshop. More research is needed to understand the influence of continuum beliefs in the social interaction between MHCP and clients. Communicating the continuity aspect of mental disorders in a workshop for MHCP could help decrease the “us” and “them” gap between professionals and people with mental health problems.

## Ethics Statement

We received an ethical approval by the University of Utrecht to conduct this research. All participants filled out an informed consent. Confidentiality and anonymity were respected in this research and participants participated voluntarily; this research was accomplished without funding and there is no conflict of interest.

## Author Contributions

All authors listed have made substantial, direct, and intellectual contribution to the work and approved it for publication.

## Conflict of Interest Statement

The authors declare that the research was conducted in the absence of any commercial or financial relationships that could be construed as a potential conflict of interest.
